# Effect of Gestational Direct-Fed Microbials Supplementation on the Metabolic Profile in Periparturient Dairy Cows

**DOI:** 10.3390/ani14202928

**Published:** 2024-10-11

**Authors:** Orlando Ramirez-Garzon, David Barber, Loreto Meneses, Martin Soust

**Affiliations:** 1 Terragen Biotech Pty Ltd., Coolum Beach, QLD 4573, Australia; msoust@me.com; 2 School of Veterinary Science, The University of Queensland, Gatton, QLD 4343, Australia; 3 DairyNEXT Nutrition Consulting Services, Marburg, QLD 4346, Australia; qlddairynutrition@hotmail.com; 4 Independent Researcher, Brisbane, QLD 4101, Australia; loreto.meneses@gmail.com; 5 Martin Soust & Co Pty Ltd., Melbourne, VIC 3146, Australia; 6 GR International Pty Ltd., Melbourne, VIC 3146, Australia

**Keywords:** dairy cow, *Lactobacillus*, metabolic profile, periparturient performance

## Abstract

**Simple Summary:**

This study at a commercial dairy farm in Queensland, Australia, evaluated the effects of a lactic acid bacteria-based direct-fed microbial (DFM) on periparturient dairy cows. A total of 150 Holstein cows were divided into control and DFM groups, with the latter receiving a basal diet supplemented with three strains of bacteria from the genus formerly known as *Lactobacillus* but recently assigned to the new *Lentilactobacillus* and *Lacticaseibacillus* genera. Blood samples were collected to assess metabolic profiles, revealing higher serum glucose, NEFA, and chloride levels in the DFM cows. The DFM cows showed higher pre- and post-calving glucose levels and greater body weight. While milk production was slightly higher in DFM cows, the milk protein percentage was higher in the control cows. Overall, DFM supplementation positively influenced the cows’ metabolic profiles, potentially enhancing milk production and body weight postpartum.

**Abstract:**

This study was conducted at a commercial dairy farm in Queensland, Australia to evaluate the effects of feeding a lactic acid bacteria-based direct-fed microbial (DFM) during gestation on the metabolic profile of periparturient dairy cows and its effects on milk production and body weight. A total of 150 multiparous Holstein cows were randomly selected based on parity (2.3) and days in milk (130 DIM) and divided into two groups of 75 cows each (control and DFM). The control cows were assigned to a basal diet consisting of a silage-based partial mixed ration (PMR), concentrate fed in the dairy twice a day, and *ad libitum* pasture. The DFM group received the same basal diet supplemented with three strains of *Lactobacillus* top-dressed in the feed. The DFM supplementation continued during both the dry period and the subsequent lactation. A subset of 82 cows (40 control and 42 DFM) were monitored during the calving season (March to July 2022) to assess the metabolic profile and postpartum performance. Blood samples were collected during the periparturient period (−4 to −2 w prepartum, around calving, and at weeks 1, 3, and 6 postpartum) to measure the levels of metabolites, enzymes, and minerals. Overall, the serum glucose, NEFA, and chloride levels were higher, while protein and urea were lower in cows supplemented with the DFM (*p* < 0.005). The pre-calving levels of glucose were higher and the total bilirubin, urea, and BHB were lower in cows supplemented with DFM than in the control (*p* < 0.05). The post-calving levels of glucose and Mg were also higher in the DFM cows than in the control cows (*p* < 0.05). Average milk production at 110 DIM was significantly higher in the DFM cows compared to control cows (*p* = 0.03). Although the total milk production over 305 days was numerically greater in the DFM cows, the difference was not statistically significant (*p* = 0.3), whereas the milk protein percentage was higher in the control cows (*p* = 0.03). The body weight of the DFM cows was greater during the periparturient period (*p* = 0.001) than that of the control cows. In the DFM cows, glucose levels had a positive correlation (r = 0.16) with milk yield, at 110 DIM, while serum total protein had a positive correlation with body weight (r = 0.32) (*p* < 0.05). In conclusion, feeding *Lactobacillus*-based DFM during gestation can positively influence the metabolic profile of periparturient cows, which, in turn, may affect the milk production and body weight of postpartum dairy cows.

## 1. Introduction

The periparturient period in dairy cows involves major metabolic and physiological changes that impact milk production and overall health [1]. This stressful period involves calving, lactation onset, immunosuppression, weight loss, dietary transitions, increased energy demands for fetal growth, and colostrum production [2,3,4]. Dry-matter intake (DMI) declines before calving and increases postpartum but often remains insufficient to meet nutrient and energy demands, leading to a negative energy balance (NEB) and nutrient deficiencies [5,6]. These imbalances disrupt carbohydrate, fat, and protein metabolism, alter enzyme activity, and increase the demand for essential minerals [7]. As a result, cows experience reduced milk yield, poorer body condition, and heightened risk of metabolic and reproductive diseases [8,9,10,11]. Key metabolites, like glucose, NEFA, BHB, triglycerides, urea, albumin, and creatinine, provide insights into a cow’s metabolic status, particularly energy balance, nutritional status, and the risk of metabolic disorders [1]. Essential minerals, such as calcium, phosphorus, and magnesium, support bone health, milk production, and energy metabolism, while potassium, sodium, and chloride regulate electrolyte balance and fluid homeostasis [12]. Changes in enzymes like aspartate-aminotransferase (AST) and creatin kinase (CK), provide insights into glucose and lipid metabolism, liver function, and muscular breakdown [13,14]. Maintaining optimal levels of these metabolites, minerals, and enzymes during the periparturient period is crucial for improving milk yield, reproductive performance, and overall health in dairy cows [13,15,16,17].

Direct-fed microbials (DFMs) are beneficial microorganisms that enhance gut microbiota, improving nutrient breakdown and absorption in dairy cows. This leads to better energy status, nutrient availability, and immune function during lactation. DFMs, especially those with *Lactobacillus* strains, can modulate the rumen environment, boosting nutrient absorption and immunity [18,19,20]. Research indicates that prepartum DFM supplements can positively impact productivity [21], health [22,23,24], and reproductive performance [25,26,27] in the postpartum period, although the precise mechanisms are not fully understood. Nocek et al. [21] found that DFM supplementation during the transition period increased DMI and milk production postpartum, with higher blood glucose and insulin levels and lower NEFA and BHB levels. Similarly, Carpinelli et al. [28] reported that periparturient yeast supplementation increased energy-corrected milk (ECM) by 3.2 kg/d, and enhanced rumen bacterial populations, although NEFA levels rose at 7 DIM (days in milk). Lehloenya et al. [29] observed that combining yeast and *Propionibacteria* strains increased 4% fat-corrected milk, milk protein yield, and glucose and insulin levels, suggesting a potential effect of DFMs on gluconeogenesis. In contrast, Boyd et al. [30] noted increased milk yield with *Lactobaccillus* and *Propionibacterium* strains during mid-lactation but no changes in serum glucose or urea. Alzahal et al. [31] found no effect on milk yield or metabolic parameters with *Enterococus* and *Sacchoromyces* supplementation during the transition period but reported improved starch digestibility. Post-calving DFM supplementation also led to increased milk yield and components in Merati’s study [32]. West et al. [33] found lower plasma urea levels, suggesting improved nitrogen efficiency in high crude-protein diets [22]. These findings collectively suggest that DFM supplementation during gestation may offer a strategic approach to support metabolic health and performance during the critical transition from gestation to early lactation, though the effects vary depending on the specific strains, dosage, and diet used for supplementation. 

In a preliminary study [34], we reported that dairy cows supplemented with DFM showed increased milk production and improved body weight, However, we did not examine the underlying mechanisms for these outcomes. Thus, in this study, we examined the effects of supplementing lactating dairy cows during gestation with a lactic acid bacteria-based DFM on (i) the cows’ metabolic profile during the periparturient period and (ii) milk production and body weight pre- and post-calving. Thus, the hypothesis of the study is that supplementing lactating dairy cows with a lactic acid bacteria-based DFM during gestation will positively influence their metabolic profile during the periparturient period, leading to improved energy balance, increased milk production, and enhanced body weight, both pre- and post-calving, compared to non-supplemented cows.

## 2. Materials and Methods

The full details of the study location, herd, and design are published in Ramirez-Garzon et al., 2024 [34].

This study was conducted from September 2021 to January 2023 at a commercial dairy farm in Harrisville, Queensland, Australia, and involved approximately 350 Holstein crossbreed cows. From this herd, 150 multiparous cows were randomly selected and divided into two groups (control and DFM, *n* = 75 each) based on parity and days in milk (DIM). The study assessed the milk performance over two consecutive lactations, with at least 50 cows per group, ensuring a 95% confidence and 80% power to detect a 2 L difference in mean milk production (pooled variance: 12 L, 95% CI: 0.60–3.40 L).

The cows were housed in a free-stall yard and managed in two groups separated by fencing for feeding and milking and with free access to water. The DFM group received a TMR diet supplemented with 10 mL/cow of a DFM (manufacturer’s recommendation), top dressed during lactation and the drying off period using a 2 L manual pressure sprayer (245 kPa maximum pressure, Aqua Systems Bunnings Warehouse, Brisbane, Australia). The DFM contained approximately 3.5 × 10^9^ CFU of a consortium of three strains of lactic acid bacteria (LAB), namely *Lentilactobacillus buchneri Lb23*; *Lacticaseibacillus casei Lz26*; and *Lacticaseibacillus paracasei T9* (Mylo^®^, Terragen Biotech, Coolum Beach, QLD, Australia). The total mixed ration (TMR) diet was formulated using available ingredients and varying pasture quality to meet milk production targets based on each lactation stage (Table 1). The TMR was provided once daily to both groups, targeting a DMI of 21 to 22 kg/cow/day, including pasture intake.

A subset of eighty-nine cows (control = 43 and DFM = 46), dried two months before the calving season (March to July 2022), were enrolled in this study to assess their metabolic profile during the periparturient period. At the start of the trial (October/2021), the cows were on average 72 ± 34 days pregnant. The periparturient cow population was 4.2 (±1.0) yr, 558.8 ± (122.3) Kg body weight, 2.75 ± (0.2) BCS, and included 50% in their second lactation, 22% in their third lactation, and 28% in their fourth lactation. The cows were allocated to separate adjacent prepartum paddocks and fed grazing pastures supplemented with a TMR formulated to meet the nutritional requirement for dry dairy cows. The DFM cows received a supplement of TMR silage with DFM, while the control cows received TMR only.

### 2.1. Blood Collection and Determination of Serum Analytes 

Blood samples were collected from each animal at 6 to 1 w before calving (−42 ± 5 d), at calving (0 ± 2 d), week 1 (7 ± 2 d), week 3 (21 ± 3 d), and week 6 (42 ± 2 d) relative to parturition. Samples were collected after milking between 7–9 a.m., before feeding via venipuncture of the coccygeal vein/artery. Ten mL of blood were collected in a vacutainer tube (BD Vacutainer, Becton Dickinson, Franklin Lakes, NJ, USA) without anticoagulant using 20-gauge × 2.54 cm needles (Becton, Dickinson). After collection, all blood samples were stored in refrigeration. Within 24 to 48 h, the blood samples were submitted for serum extraction and biochemical analysis to the Veterinary Laboratory Services from the University of Queensland (Gatton, Australia). The serum was extracted by centrifugation (1500× *g* for 5 min) and stored in refrigeration. The serum concentrations of metabolites (albumin, glucose, total protein, total bilirubin, triglycerides, urea, non-esterified fatty acids (NEFA), and beta-hydroxybutyrate (BHB) enzyme activities (aspartate aminotransferase (AST), creatin kinase (CK), gamma-glutamyl transferase (GGT), and minerals (Ca, P, Mg, Na, K, and Cl) were analyzed using an Olympus AU400 Clinical Chemistry Analyzer (Beckman Coulter, Brea, CA, USA).

### 2.2. Milk, Fat, and Protein Yield 

The cows were milked twice daily at 0400 and 1500 h, with the individual milk yields electronically recorded via DairyPlan C21A software (GEA Farm Technologies, Germany). Weekly averages were used for statistical analysis. Composite milk samples were taken every 6–8 weeks as part of the herd’s udder health and mastitis control program, and to evaluate milk fat and milk protein percentages. The samples were collected using automated milk meters (GEA Westfalia, Melbourne, Australia), placed in vials with a preservative (Bromoponol, Novachem, Heidelberg, Australia), and sent to a commercial lab (Dairy Express Herd Recording Service, Armidale, NSW, Australia) for analysis. Both the milk yield and the milk components were assessed during the study period (March 2022–January 2023).

### 2.3. Body Condition Score and Body Weight

The body condition score (BCS) was determined for all study cows at blood collection time by a single investigator using a 1 to 5 score with a quarter-point system (1—emaciated, 5—obese) [35]. Cow weights were recorded automatically after each milking session using a walk-over weigh unit (WOW; Datamars, New Zealand).

### 2.4. Data Collection

Milk production was collected from the farm’s DairyPlan C21A (GEA Farm Technologies, Germany) records. The collected milk production data included weekly milk yield, average milk yield at 30, 50, 90, and 110 DIM), total milk yield (305 DIM), peak milk (L), peak milk DIM, predicted 305 day milk yield, total milk fat (Kg), total milk protein (Kg), as well as average milk fat (%), and average milk protein (%).

### 2.5. Statistical Analysis

The statistical analysis was performed using R 4.4.0 software. A descriptive analysis of the data was conducted to validate the dataset and identify potential outliers using Cook’s distance. For comparisons between the treatment and control groups or between the different measurement periods (pre-calving, calving, and 1 week, 3 weeks, and 6 weeks post-calving) a two-sample *t*-test was used to compare means when the data met the assumptions of normality and equality of variances, as assessed by the Shapiro–Wilk test and Levene’s test, respectively. The Mann–Whitney test was used to detect differences between medians when the data were not normally distributed by the Shapiro–Wilk test.

All significance tests were conducted at an alpha level of 0.05, and missing observations were excluded from the analysis. To identify associations between variables, Pearson’s correlations were used with a significance level of 0.05. For categorical variables, polychoric correlations were used to assess associations.

## 3. Results

Seven cows were removed from the study due to farm management decisions unrelated to the treatment, with three cows from the control group and four cows from the DFM group.

### 3.1. Metabolic Profile 

**Figure 1:**
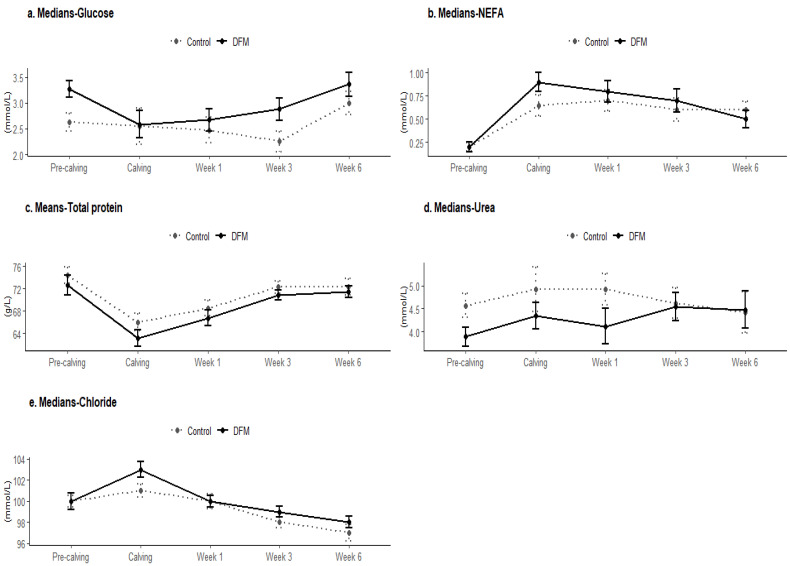
Serum glucose, total protein, urea, NEFA, and chloride profiles of control and DFM cows during periparturient period (*p* < 0.05). Means (±SD) are presented for normally distributed data (*t*-test), while medians (±SD) are shown for non-normal distributions (Mann–Whitney test).

**Figure 2:**
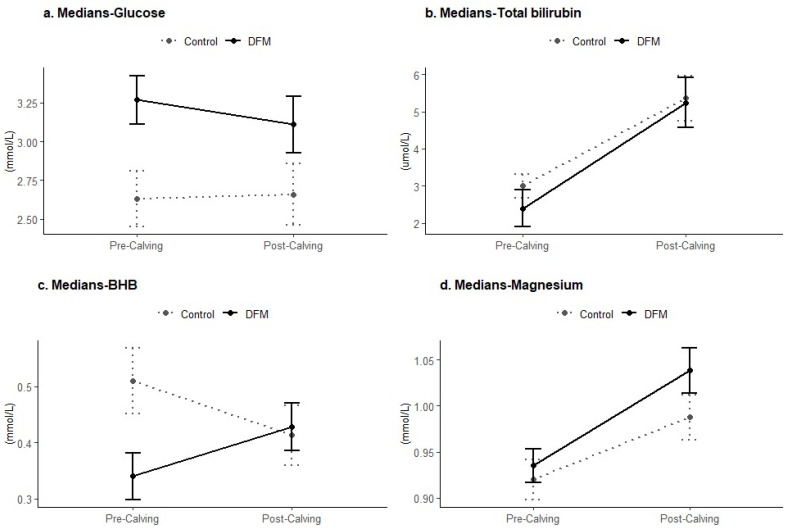
Pre-calving and post-calving serum profiles of glucose, total bilirubin, beta-hydroxybutyrate (BHB), and magnesium of control and DFM cows (*p* < 0.05).

Overall, the serum glucose levels were significantly higher in the DFM cows compared to the control cows during both the pre-calving (*p* < 0.01) and post-calving (*p* < 0.05) periods. Circulating glucose concentrations were higher in both groups during the pre-calving period, although the DFM cows had a significantly higher serum concentration (*p* < 0.01) (Table 2). At calving, glucose levels dropped in both groups. In the post-calving period, glucose levels in the DFM cows progressively increased at 1 week, 3 weeks, and 6 weeks. Conversely, in the control cows, glucose levels tended to decrease at 1 week and 3 weeks before showing an increase at 6 weeks (Figure 1a and Figure 2a). 

Circulating levels of NEFA were also higher in the DFM cows (*p* < 0.05), but no significant differences were observed between the pre-calving and post-calving periods (*p* > 0.05) (Table 2). Pre-calving NEFA concentrations were similar in both groups, then increased at calving, with the DFM cows exhibiting higher concentrations than the control cows (*p* < 0.05). After calving, NEFA concentrations in the DFM cows consistently decreased at 1 week, 3 weeks, and 6 weeks. In contrast, the control cows showed an increase in the NEFA concentration at week 1, followed by a decrease at 3 weeks and 6 weeks (Figure 1b).

While there were no significant differences in overall BHB and total bilirubin levels (*p* > 0.05) (Table 2), the DFM cows exhibited lower concentrations in both during the pre-calving period (*p* < 0.05) (Figure 2b,c). Additionally, no significant differences in triglycerides and bile acids were found between the DFM and control cows (*p* > 0.05). 

Total protein and urea blood levels were consistently lower in the DFM cows compared to the control cows (*p* < 0.01), with the DFM cows showing lower urea levels before calving (*p* < 0.01). Pre-calving concentrations of total protein were higher in both groups compared to the concentrations at calving. After calving, total protein concentrations in both groups increased at 1 week and 3 weeks, maintaining their levels at 6 weeks. Urea concentrations were lower before calving in both groups compared to calving levels. After calving, the DFM cows showed a drop in their urea concentration during the first week, followed by a rise at 3 weeks, and maintained their levels at 6 weeks. In contrast, the control cows maintained their urea concentrations during the first week, then showed a drop at 3 weeks, and maintained their levels at 6 weeks (Figure 1c,d). There were no significant differences in albumin and creatinine serum levels between the two groups (*p* > 0.05) (Table 2). 

Furthermore, there were no significant differences in circulating enzyme levels (CK, GGT, AST) between the DFM and control cows (*p* > 0.05). Serum concentrations of Ca, P, Na, and K were not significantly different between the groups (*p* > 0.05). However, the DFM cows had higher overall levels of Cl (*p* < 0.05, Figure 1e) and higher post-calving serum Mg levels compared to the control (*p* < 0.05, Figure 2d). 

### 3.2. Productive Performance 

The effect of supplementing DFMs on milk yield, milk components, body weight, and interactions are presented in Table 3. The DFM cows tended to produce more milk throughout the entire lactation, yielding 4.2% more milk than the control cows at 305 DIMs. Additionally, the DFM cows had a 5.6% higher average daily milk yield (*p* = 0.07) during the study period (March 2022–January 2023). The average milk yield ranged between 2.7 to 7.3% higher during the first 110 DIMs (*p* = 0.03). The DFM cows also achieved a higher peak milk production (+2.7 L/cow *p* = 0.02) and achieved peak milk 7 days earlier (*p* = 0.89) than the control cows. There was no difference between groups for milk fat (%), while milk protein (%) was higher in the control cows compared to the DFM cows (*p* = 0.03). 

There was no significant difference in body condition score (BCS) pre-calving or post-calving between the DFM and control cows. However, the DFM cows showed a greater overall body weight during the periparturient period (*p* = 0.001) and during the first 6 weeks postpartum (*p* = 0.02).

**Figure 3:**
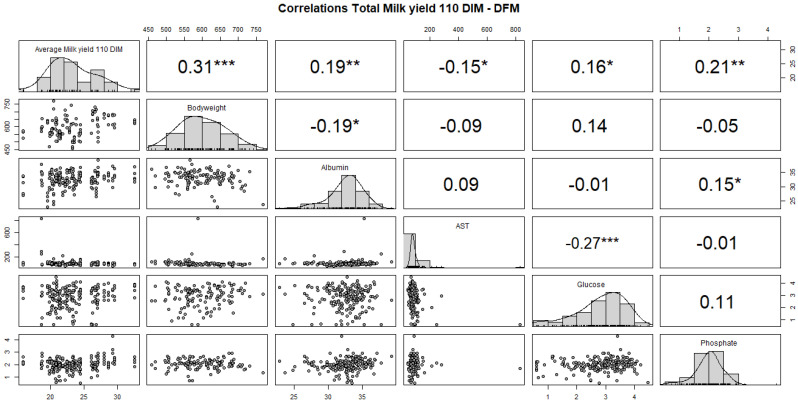
Correlation of total milk 110 DIMs production of DFM cows. Aspartate aminotransferase (AST). *p*-Value * 0.05; ** 0.01; *** < 0.01.

In the DFM cows, the total milk yield at 110 DIMs showed a significant positive correlation with glucose levels (r = 0.16, *p* < 0.05), body weight (r = 0.31, *p* < 0.001), albumin (r = 0.19, *p* < 0.05), and phosphate (r = 0.21, *p* < 0.01) and a negative correlation with AST (r= −0.15, *p* < 0.05) with milk yield at 110 DIMs (Figure 3).

**Figure 4:**
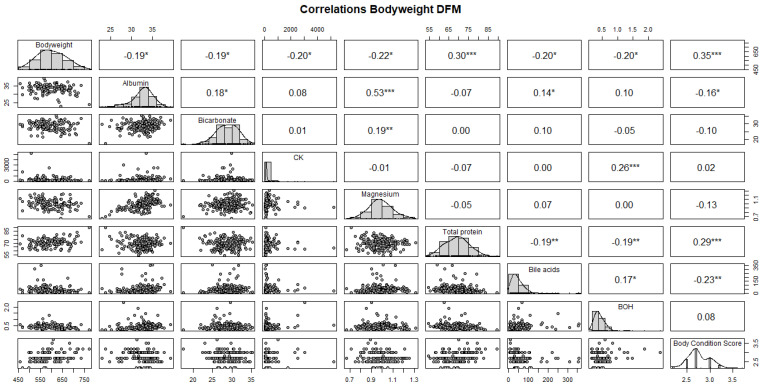
Correlations of body weight in DFM cows. Creatin kinase (CK), beta-hydroxybutyrate (BHB). *p*-value * 0.05; ** 0.01; *** < 0.01.

Body weight in the DFM cows has a positive correlation with total protein (r = 0.3; *p* < 0.001) and a negative correlation with albumin (r = −0.19), bicarbonate (r = −0.19), CK (r = −0.2), Mg (r = −0.22), bile acids (r = −0.2), and BHB (r = −0.2) (*p* < 0.05) (Figure 4).

## 4. Discussion

In this study, supplementing cows with DFMs during gestation resulted in specific changes in the metabolic profile in periparturient cows. DFM supplementation was associated with higher serum glucose and NEFA concentration and lower urea and protein levels in DFM cows compared to untreated cows. In the DFM cows, the serum levels of glucose were correlated with milk production at 110 DIMs. Total protein was associated with overall body weight postpartum. Cows supplemented with DFMs showed lower levels of total bilirubin and BHB pre-calving and higher levels of Mg post-calving.

The blood glucose concentration is a key indicator of energy balance in dairy cows [36]. Elevated glucose levels indicate increased energy derived from dietary carbohydrates, which helps meet the high energy demands of the periparturient period for fetal growth and milk production [3,5,37].During mid-gestation, the placenta consumes approximately 80% of the glucose from maternal blood, and from mid-to-late gestation, the placenta’s glucose demand increases six-fold to support the ten-fold increase in fetal mass [38,39]. In this study, supplementing gestating cows with DFMs led to increased serum glucose levels before calving. However, the implications of these elevated glucose levels on the offspring were not assessed.

Although feed intake and ruminal microbiome were not assessed in this study, we can speculate that DFM-treated cows displayed better digestibility based on higher glucose levels before calving and postpartum. The ruminal digestion of starch and fiber produces lactic acid and volatile fatty acids (VFAs), predominantly acetate, propionate, and butyrate, through microbial fermentation [40]. Ruminants primarily meet their glucose needs through gluconeogenesis in the liver, using glucose precursors (VFAs) absorbed after diet fermentation and digestion [41]. Propionate acts as a primary precursor for hepatic gluconeogenesis, which, when released into the circulation, provides almost 80% of the glucose to lactating cows, enhancing lactose synthesis in the mammary gland and, consequently, increasing milk yield [42,43]. Post-calving, the glucose levels in the DFM cows increased consistently at 1, 3, and 6 weeks. This increase positively correlated with milk yield at 110 DIMs (r = 0.16, *p* < 0.05), explaining the observed differences in average milk yield in the DFM cows (24.8 L vs. 23.4 L, *p* = 0.07) and the higher peak milk (34.5 L vs. 31.9 L, *p* = 0.02) compared to the control cows. Although some studies have not shown any beneficial effect on glucose levels and milk yield with DFM supplementation (*Saccharomyces* and *Entreorcoccus*) during the transition period [31], our study found that DFM cows yielded 6.4% more milk during the first 110 DIMs (*p* = 0.03) compared to the control cows. This increase is consistent with other studies that reported a 4.5 to 9.5% increase in milk production during the first 10 weeks of lactation. These increases have been associated with higher blood glucose and insulin levels postpartum in DFM-supplemented cows (yeast and *Enterococcus*) during the transition period [21,44].

It is considered ideal for cows to have a moderate BCS (>2.5 and <3.0) at the beginning of the transition period [10] due to the physiological weight loss and decline in body condition that occurs during early lactation [45]. In this study, the DFM-treated cows had a higher BCS (*p* > 0.05) and body weight (*p* < 0.05) pre-calving compared to the controls. Post-calving, both groups experienced a drop in BCS and body weight during the first six weeks postpartum, indicating increased fat mobilization with elevated NEFA and BHB levels [10]. 

In this study, DFM-supplemented cows exhibited higher NEFA levels (*p* < 0.05) with no significant differences in serum levels between the pre-calving (0.2 mmol/L) and post-calving (0.7 mmol/L) periods. These NEFAs are utilized for milk fat synthesis or as an energy source for milk production [11]. Although other studies [46,47] have reported a negative association between periparturient NEFA levels (0.5–0.7 mmol/L) with milk yield, we did not find any significant correlation between NEFA levels and milk production. Additionally, milk fat (%) was similar between the DFM and control groups. Although excessive NEFA can lead to the accumulation of triglycerides in the liver, leading to fatty liver [48], no differences were detected in liver enzymes (AST and GGT), which are indicative of liver damage [13,49]), nor in the triglyceride levels between the DFM and control cows. These findings suggest that, despite higher NEFA levels in DFM-supplemented cows, no adverse effects on milk production were observed throughout the study. The lack of effect on milk yield could be due to insufficient statistical power or sample size to detect a significant difference. Another possible explanation is that cows with higher NEFA levels may experience short-term productivity losses but adapt to the energy demands, eventually increasing milk production as lactation progresses [46,50]. Further studies with larger sample sizes and controlled experiments focusing on long-term metabolic adaptations to elevated NEFA levels would help to clarify the role of NEFA in milk production across different lactation stages.

BHB levels in both groups ranged between 0.3 and 0.5 mmol/L, and DFM cows had significantly lower pre-calving BHB levels (*p* < 0.05) compared to control cows, suggesting reduced fatty acid mobilization in the DFM cows [44]. These levels are lower than the levels associated with subclinical ketosis (SCK) (>1.2–14.4 mmol/L) [3], which is typically accompanied by a milk fat content over 5% and milk protein levels lower than 2.9% [37]. In this study, DFM cows exhibited a higher BCS before calving compared to controls. Similarly, Oetzel et al. [23] found that DFM-supplemented cows increased the BCS of dry cows by 0.25 units, increased milk yield by 0.8 kg/d, and a rise in the BHB concentration. Additionally, BHB can serve as an energy substrate for various tissues, sparing glucose for lactose production [51]. Thus, DFM supplementation in cows during gestation can lead to higher BCS and body weight with increased NEFA levels post-calving directed towards milk production. However, it does not adversely affect productivity or liver health, and BHB levels remain below those associated with subclinical ketosis.

Protein metabolism can be evaluated by the determination of albumin, total serum proteins, and urea in blood [41]. In our study, total protein and urea concentrations (*p* < 0.01) were lower in the DFM cows before calving and during the first six weeks postpartum, which reflects efficient nitrogen capture in the rumen, avoiding wastage of nitrogen [41]. Cows supplemented with DFMs during gestation experienced less pronounced body weight loss postpartum and, therefore, are likely to have recovered more quickly from the negative energy balance.

The level of blood urea nitrogen serves as an indicator of protein utilization efficiency, degradability of protein in the rumen, and post-ruminal protein intake [52,53]. The concentration of blood urea-N is related to the absorption of ammonia from the rumen and/or the deamination of amino acids not deposited in the tissue [54]. In our study, we observed lower levels of urea during early postpartum in the DFM-treated cows compared to the control cows, which indicates that the protein ingested is being used more efficiently by the body rather than being broken down into ammonia and converted to urea. DFMs can improve rumen microbial activity, leading to better protein degradation and utilization in the rumen [55,56]. If the DFM-treated cows are utilizing protein more efficiently, they are likely converting a greater proportion of their dietary protein into muscle and other tissues rather than excreting it as urea. This could explain the observed improvement in overall body weight in the DFM-treated group.

The increase in Mg and Cl levels in periparturient cows supplemented with DFMs could be linked to higher dry-matter intake (DMI) and increased milk production. These electrolytes are essential for maintaining fluid balance, nerve function, and overall metabolic activity, which are crucial during the transition to lactation [57,58]. The elevated levels of Mg and Cl may indicate that the cows are better able to meet the nutritional demands of early lactation, leading to improved milk yield.

Overall, this study supports the previous findings that supplementing dairy cows with DFMs leads to increased milk yield during early lactation and liveweight [34]. Specifically, DFM-treated cows showed higher serum glucose levels, correlating with increased milk production, and exhibited higher body weight and body condition scores pre-calving. Post-calving, DFM cows had elevated glucose and NEFA levels, suggesting enhanced energy utilization for milk production and better overall metabolic efficiency.

It is worth emphasizing that the effect of DFM supplements is influenced by several factors, including the microbial strains used, the dosage, timing, and duration of supplementation, diet, breed, or farm-specific conditions. In this study, a key limitation was the lack of individual dry-matter intake (DMI) assessment, which should be considered when interpreting the results. Despite this, the metabolic profile findings remain valuable, offering insights into cows’ physiological adaptations during the periparturient period and also providing useful metabolic trends that can guide farm management practices, even in the absence of precise DMI data. Although these limitations exist, this study serves as a valuable basis for generating hypotheses to be tested in more controlled settings, where individual intake can be monitored. The findings in this study may be specific to the lactic acid bacteria strains used and the farming system in which they were examined. Further research is needed to explore the effects of different DFM strains and combinations under various farming conditions and with different cow breeds to better understand their potential benefits and optimize their use in dairy production.

## 5. Conclusions

Based on the findings of this study, supplementing dairy cows with lactic acid bacteria-based DFMs during gestation positively influenced the metabolic profile of periparturient cows, leading to improved milk production and increased body weight postpartum. These results suggest that DFMs can effectively boost the energy status and milk yield in dairy cows, though further research is needed to optimize DFM use and assess the long-term impacts on offspring.

## Figures and Tables

**Table 1:** Ingredient and chemical composition of diet (%, DFM basis).

Ingredient	Mean	Min	Max
Grain (Barley, wheat, sorghum)	19.3	15.8	27.7
Protein Meal (canola meal, soybean meal)	7.7	0	17.4
Byproducts (Flour bread, carrots, sweet corn waste, chickpea millrun)	14.3	3.1	35.3
Lucerne Hay	5.8	3.3	9.9
Silage (corn, barley, oats, soybean)	34.7	14.8	49.2
Pasture (Kikuyu, ryegrass)	15.3	0	43
Bypass Fat	0.6	0	1.9
Minerals (macro minerals, trace minerals premix, urea, mycotoxin binder)	2.4	1.2	3.5
Diet Composition			
Crude Protein (CP, %)	16.9	13.8	22.8
Neutral Detergent Fiber (NDF, %)	35.9	31.2	40
Acid Detergent Fiber (ADF, %)	23.2	19.3	26.2
Non-Fibrous Carbohydrate (NFC, %)	34.3	28.1	40.2
Fat (%)	4.4	3.6	5.6
MJ ME/Kg DM	9.5	8.6	10.8
Starch (%)	23	20.1	26.5

**Table 2:** Metabolic profile of control and DFM cows during the peripartum period (±SD).

	Control Overall	Pre-Calving	Post-Calving	DFM Overall	Pre-Calving	Post-Calving
	Metabolites					
Glucose (mmol/L)	2.6 (±0.9)	2.6 (±0.7)	2.7 (±0.8)	2.9 (±0.9) **	3.3 (±0.6) **	3.1 (±0.7) *
NEFA (mmol/L)	0.6 (±0.4)	0.2 (±0.2)	0.7 (±0.4)	0.7 (±0.5) *	0.2 (±0.2)	0.7 (±0.4)
BHB (mmol/L)	0.4 (±0.2)	0.5 (±0.2) *	0.4 (±0.2)	0.4 (±0.3)	0.3 (±0.1)	0.4 (±0.2)
Total bilirubin (µmol/L)	4.9 (±3.1)	3.0 (±1.1) *	5.8 (±2.3)	4.8 (±3.9)	2.4 (±1.8)	5.4 (±2.5)
Triglycerides (mmol/L)	0.1 (±0.1)	0.2 (±0.1)	0.1 (±0.1)	0.1 (±0.1)	0.2 (±0.1)	0.1 (±0.1)
Bile acids (µmol/L)	35.3 (±54.1)	16.1 (±9.1)	48.4 (±62.4)	38.1 (±53.9)	16.3 (±17.2)	48.1 (±44.1)
Total Protein (g/L)	70.9 (±5.9) **	74.4 (±5.3)	71.0 (±4.6)	69.1(±6.1)	72.6 (±6.6)	70.1 (±3.4)
Urea (mmol/L)	4.7 (±1.3) **	4.6 (±0.9) **	4.6 (±1.1)	4.1 (±1.2)	3.8 (±0.8)	4.3 (±1.0)
Creatinine (µmol/L)	78.1 (±16.3)	95.8 (±14.1)	72.3 (±10.7)	76.8 (±16.4)	90.2 (±12.6)	70.3 (±9.5)
Albumin (g/L)	32.6 (±2.6)	31.2 (±2.0)	32.9 (±2.6)	33.0 (±2.6)	31.8 (±2.4)	34.0 (±2.4)
Enzymes						
CK (U/L)	149.5 (±270.3)	136.2 (±125.8)	174.8 (±137.7)	137.5 (±553.7)	112.9 (±857.9)	154.9 (±274.4)
GGT (U/L)	16.4 (±9.8)	13.5 (±7.3)	17.8 (±9.7)	15.1 (±6.4)	11.1 (±8.2)	16.2 (±4.5)
AST (U/L)	84.5 (±18.9)	76.8 (±18.1)	89.4 (±15.0)	83.4 (±60.9)	73.7 (±23.0)	81.4 (±77.2)
Minerals						
Ca (mmol/L)	2.3 (±0.2)	2.3 (±0.1)	2.3 (±0.2)	2.3 (±0.2)	2.3 (±0.1)	2.3 (±0.2)
Phosphate (mmol/L)	2.0 (±0.5)	2.1 (±0.5)	2.2 (±0.3)	2.0 (±0.5)	2.0 (±0.5)	2.1 (±0.3)
Mg (mmol/L)	1.0 (±0.1)	0.9 (±0.1)	1.0 (±0.1)	1.0 (±0.1)	0.9 (±0.1)	1.0 (±0.1) *
Na (mmol/L)	139 (±2.6)	140.8 (±2.3)	138.2 (±1.5)	139 (±2.8)	140.2 (±3.2)	138.5 (±2.0)
K (mmol/L)	5.3 (±1.1)	4.9 (±1.6)	5.4 (±0.7)	5.2 (±1.0)	4.8 (±0.7)	5.4 (±1.0)
Cl (mmol/L)	99 (±2.8)	100.1 (±2.1)	98.1 (±1.9)	100 (±2.8) *	100.8 (±2.9)	98.8 (±1.8)

Non-esterified fatty acids (NEFA), beta-hydroxybutyrate (BHB), creatin kinase (CK), gamma-glutamyl transferase (GGT), and aspartate aminotransferase (AST). *p* < 0.05 *: *p* < 0.01 **.

**Table 3:** Productive performance of control and DFM cows during postpartum (±SD).

	Control	DFM	*p*-Value
Total milk production (L)/cow	7670.0 (±1188.2)	7992.6(±1507.8)	0.4
Average milk/day (L)/cow	23.4 (±3.1)	24.8 (±3.8)	0.07
Average milk yield 30 DIM (L)/cow	28.6 (±5.1)	30.0 (±5.9)	0.26
Average milk yield 50 DIM (L)/cow	29.0 (±4.8)	31.3(±5.8)	0.05
Average milk yield 90 DIM (L)/cow	28.7 (±4.4)	29.5 (±4.7)	0.42
Average milk yield 110 DIM (L)/cow	21.8 (±2.9)	23.3 (±3.5) *	0.03
Total milk 305 DIM (L)/cow	7222.6 (±1107.7)	7570 (±1167.7)	0.3
Peak Milk (L)	31.9 (±4.6)	34.5 (±5.7) *	0.02
Peak DIM	71 (±36.4)	64 (±41.8)	0.89
Total milk fat (kg)	287 (±52.4)	290 (±58.5)	0.8
Average milk fat (%)	3.7 (±0.5)	3.7 (±0.6)	0.8
Total milk Protein (kg)	264.8 (±41.1)	266 (±50.4)	0.9
Average milk protein (%)	3.5 (±0.2) *	3.3 (±0.2)	0.03
BW (Periparturient) (kg)	575 (±56.2)	599.3 (±63.5) *	0.001
BW Pre-calving (kg)	602.3 (±61.2)	614.2 (±70.7)	0.4
BW Post-calving (kg)	567.3 (±52.0)	598.9 (±58.8) *	0.02
BCS (Periparturient)	2.7 (±0.3)	2.7 (±0.3)	0.8
BCS Pre-calving	3.0 (±0.3)	3.0 (±0.3)	0.2
BCS Post-calving	2.7 (±0.2)	2.7 (±0.2)	0.5

DIM (days in milk), BW (body weight), and BCS (body condition score); * *p* < 0.05 significant difference.

## Data Availability

Data will be available upon request by contacting the author.
